# Atypical melanocytic proliferation with halo reaction in a 5-year-old Filipino boy with vitiligo: A case report

**DOI:** 10.1016/j.jdcr.2025.01.011

**Published:** 2025-02-04

**Authors:** Alyssa Felsophie S. Silor, Jerson Jerick N. Taguibao, Carmela Augusta F. Dayrit-Castro, Eileen Liesl A. Cubillan

**Affiliations:** Department of Dermatology, University of the Philippines–Philippine General Hospital, Manila, Philippines

**Keywords:** case report, halo reaction, intermediate melanocytic tumors, melanocytic tumor of uncertain malignant potential, melanocytomas

## Introduction

In the pediatric population, melanoma is rare.^1^ The majority of melanoma in children is acquired from sun exposure and it has a less aggressive behavior in children.^2^ The incidence of melanoma increases with age. Clinically, a modified ABCDE (Amelanotic, Bleeding, Color uniformity, De novo, and Evolution) are clues in the diagnosis of melanoma.^3^ However, if histopathologic findings are equivocal, one should think twice before clinching the diagnosis of melanoma especially in children.

## Case report

**Fig 1:**
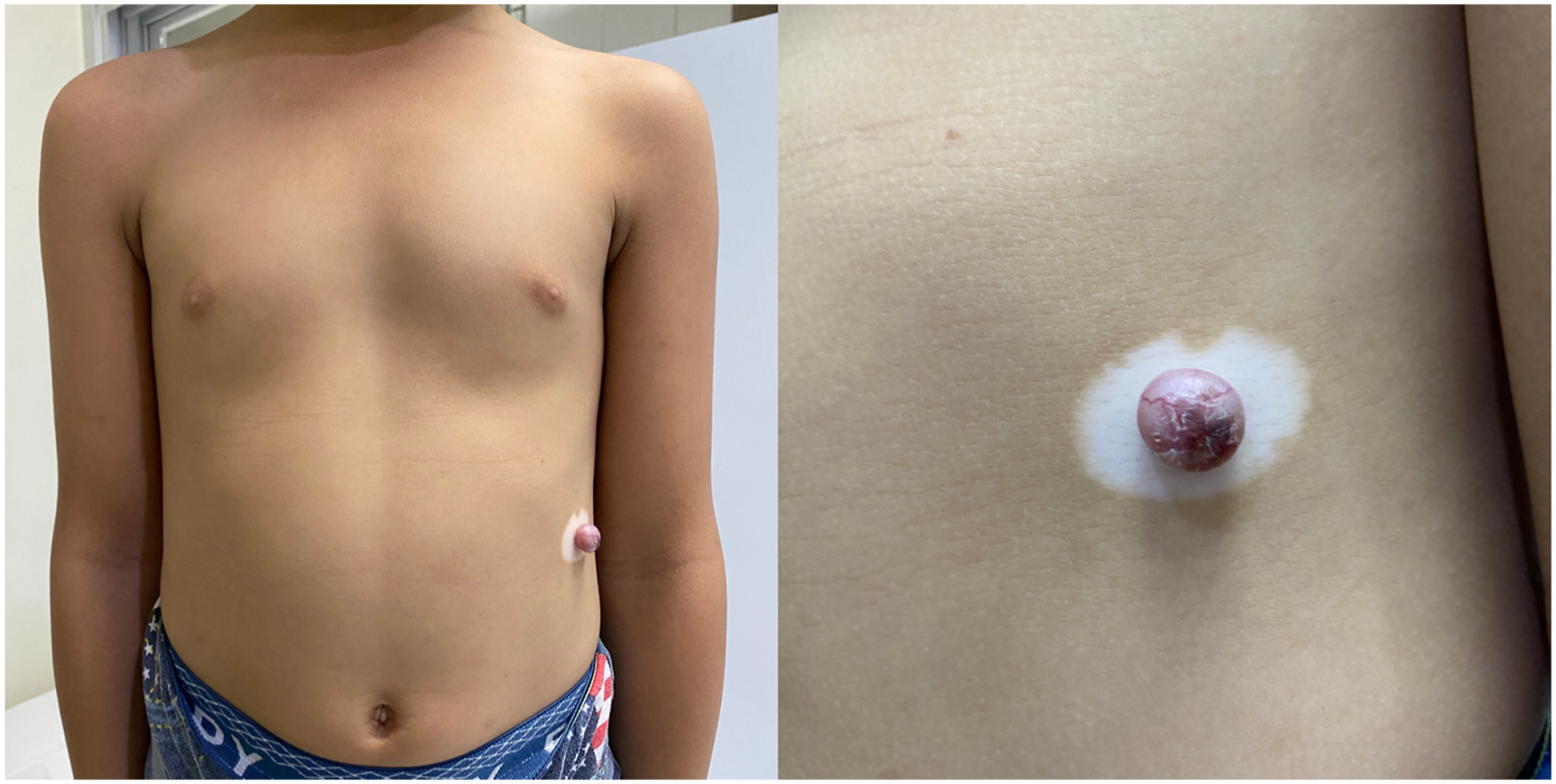
Solitary round, erythematous and slightly translucent nodule on a white base on the upper quadrant of the left side of the abdomen.

**Fig 2:**
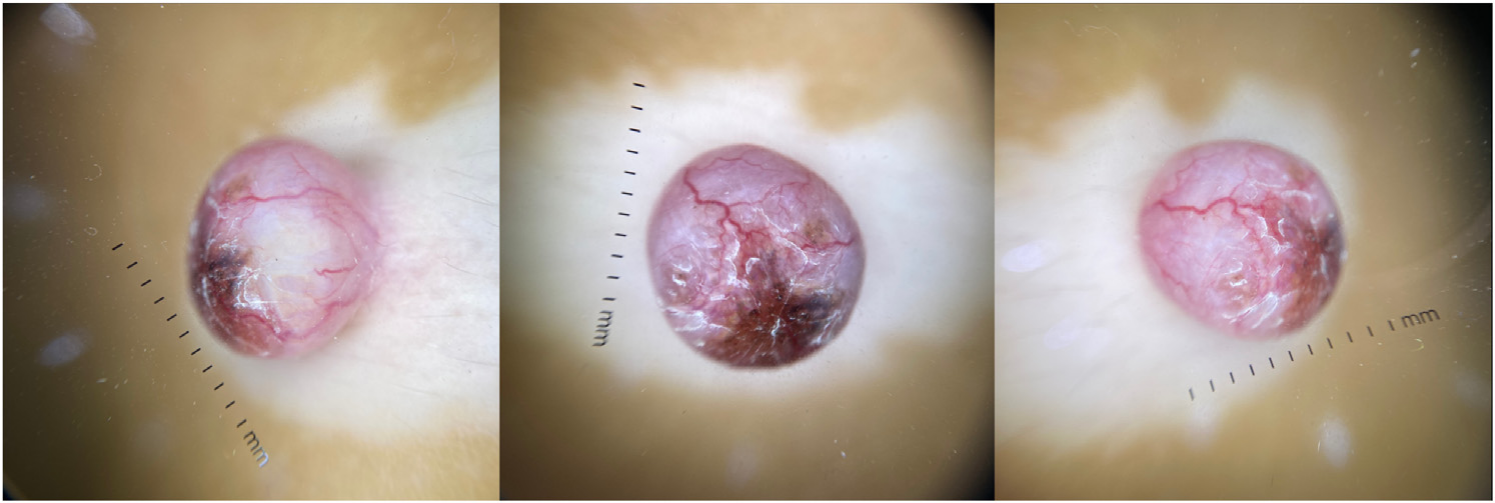
Dermatoscopy: arborizing vessels and a brown pigmented tip.

**Fig 3:**
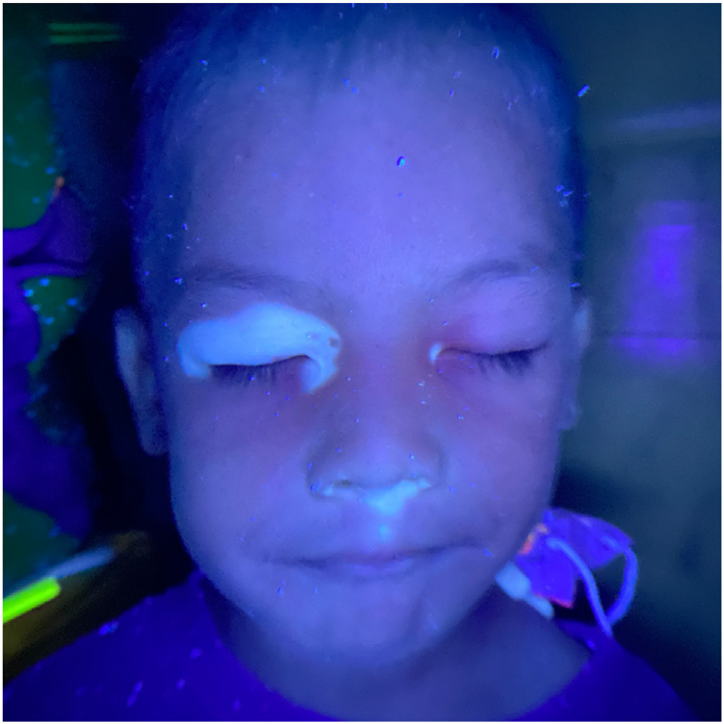
Wood’s lamp examination: blue-white fluorescence of the white macules and patches.

A 5-year-old Filipino boy from Manila, Philippines consulted at the outpatient department with a solitary round, smooth-surfaced, soft, erythematous, and slightly translucent, nontender nodule measuring 1.0 × 1.0 × 0.5 cm with a surrounding depigmented halo on the upper quadrant of the left side of the abdomen (Fig 1). This initially presented as a black macule at birth that gradually increased in size into a nodule that was occasionally pruritic and was not painful with no spontaneous bleeding. Dermatoscopic analysis of the nodule exhibited arborizing vessels and a brown pigmented tip (Fig 2). The surrounding depigmented patch presented at 1 year of life and similar vitiliginous patches appeared on the periorbital area of the right eye, medial canthus of the left eye, columella, philtrum, and dorsum of the right foot over 2 years (Fig 3). A review of systems was unremarkable. The patient is a healthy, young male with no comorbidities, no frequent exposure to ionizing radiation, an only child of a healthy nonconsanguineous couple, with a family history of vitiligo, breast cancer, and prostate cancer. Significant sun exposure includes a history of sunburns and biking for around 3 hours during midday without any photoprotective measures.

**Fig 4:**
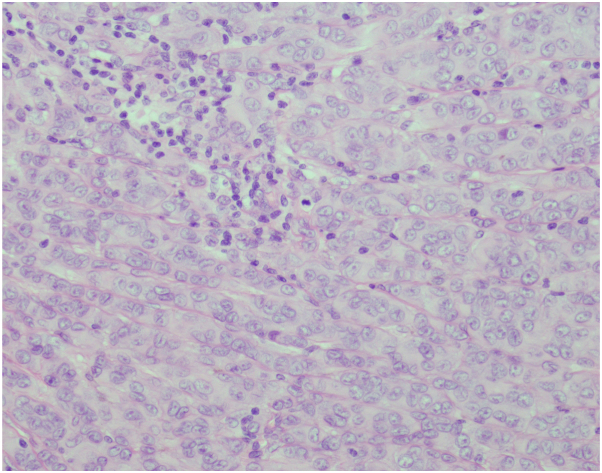
Dermal proliferation of large and atypical, some epithelioid cells, with hyperchromatic nuclei, and few scattered mitotic figures, some in cords and strands insinuating between collagen bundles (Hematoxylin-eosin stain; original magnification: ×20).

An excision biopsy under local anesthesia was done. Histopathologic examination showed a dermal proliferation of large and atypical, some epithelioid cells, with hyperchromatic nuclei, and few scattered mitotic figures, some in cords and strands insinuating between collagen bundles. Melanin pigment was seen within areas of the proliferation. There is a moderately dense interstitial infiltrate composed of lymphocytes in the dermis (Fig 4). A Melan-A stain showed strong cytoplasmic staining in majority of the proliferation, whereas a p16 stain showed strong nuclear and cytoplasmic staining. The final histopathologic diagnosis is a melanocytic tumor of uncertain malignant potential with halo reaction.

Melanocytic markers such as SOX-10, S-100, and HMB-45 showed staining in the tumor cells. A slide review done by the Pathology service considered the immunohistochemical findings compatible with a malignant melanoma. After expert evaluation and a multidisciplinary conference, the diagnosis of melanocytic tumor of uncertain malignant potential was maintained and the patient was referred to Pediatric Surgery for a wide excision of the surgical bed. Frozen section revealed no residual tumor. Close follow-up and monitoring every 6 months for the next 5 years was advised and no recurrence was noted at 6 and 12-month postexcision.

## Discussion

Melanocytic lesions are not always dichotomous. Cytoarchitectural atypia that goes beyond the definition of a benign nevus but falls short of a melanoma should prompt the clinician to consider intermediate melanocytic tumors such as melanocytomas. In the most recent WHO Classification of Skin Tumors, melanocytomas have multiple driver mutations that carry the risk of malignant transformation.^4^ Melanocytomas can be pigmented epithelioid, WNT-inactivated, BAP1-inactivated, non-spitz or not otherwise specified, and spitz. There is a 2-step pathogenesis of melanocytomas: the initiating genetic abnormality, usually BRAF/NRAS, leading to the development of a nevus, and the subset-defining genetic abnormality leading to a melanocytoma. The atypical melanocytic proliferation (melanocytic tumor of uncertain malignant potential) in this case can be categorized under melanocytoma.

Diagnosing childhood and adolescent melanoma can be challenging because they do not present with the classical features of adult melanomas. They usually present as an amelanotic, papulonodular lesion without color variegation that have arisen de novo.^5^ Majority of the young patients have a family history of atypical nevus or melanoma, which point to a genetic source. Recent data showed that UV radiation still has a role in childhood and adolescent melanoma as in this case.

Melanocytomas are more common in females with a wide age distribution. Sites of predilection include the trunk and extremities,^2^ similar to melanoma, and as seen in this patient. Evidence regarding the optimal excision margins is lacking. It is advisable to strike a balance between the morbidity of wider margins and the adequacy of treatment of ambiguous melanocytic lesions thus suggesting a safe range of 5 to 10 mm clinical margins and at least 2 mm histologic margins.^6^ Patients with melanocytomas have a favorable prognosis with low malignant potential and low chances of local recurrence or progression.^7^

On histopathology, similar in this case, a pigmented epithelioid melanocytoma can present as an intradermal melanocytic proliferation with a mixture of epithelioid and dendritic melanocytes with large nuclei and nucleoli, which are heavily pigmented with many interspersed melanophages.^8^ The Melan-A stain result cannot help distinguish a benign nevi from melanoma, whereas the p16 stain supports a more benign condition as protein function is not silenced nor lost.

The depigmented patch or halo reaction on the base of the lesion is likely secondary to the immune response of cytotoxic CD8^+^ T cells against any melanocytic tumor and the surrounding normal melanocytes experience the collateral damage.^9^ Misdiagnosis of a benign melanocytic tumor as melanoma is commonly influenced by halo reactions due to inflammatory cells inducing cytologic atypia.

A high index of suspicion is warranted when evaluating melanocytic tumors. The tumor must be perfectly normal to be benign in the elderly, whereas it should be perfectly atypical to be melanoma in children. Routine genetic testing is not recommended, but referral to Genetics is warranted if there is significant family history.^1^ Differentiation between benign and malignant melanocytic neoplasms is possible with the aid of immunohistochemistry and molecular testing. However, these are only adjuncts to clinical and expert dermatopathologic examination. Only the course of the patient will determine the final diagnosis.^10^

Given the limited evidence and cases of intermediate melanocytic tumors, appropriate management needs to weigh the risks between overly aggressive intervention and watchful waiting. For this 5-year-old patient, a long-term clinical follow-up is the only true evidence to clarify the melanocytic tumor’s biologic behavior.

## Conflicts of interest

None disclosed.
